# Study of Women, Infant feeding, and Type 2 diabetes mellitus after GDM pregnancy (SWIFT), a prospective cohort study: methodology and design

**DOI:** 10.1186/1471-2458-11-952

**Published:** 2011-12-23

**Authors:** Erica P Gunderson, Susana L Matias, Shanta R Hurston, Kathryn G Dewey, Assiamira Ferrara, Charles P Quesenberry, Joan C Lo, Barbara Sternfeld, Joseph V Selby

**Affiliations:** 1 Division of Research, Kaiser Permanente Northern California, 2000 Broadway, Oakland, CA 94612-2304, USA; 2 Department of Nutrition, University of California, One Shields Ave, Davis, CA 95616, USA; 3 PCORI (at interim location), 1701 Pennsylvania Ave., NW, #300, Washington, DC 20016, USA

## Abstract

**Background:**

Women with history of gestational diabetes mellitus (GDM) are at higher risk of developing type 2 diabetes within 5 years after delivery. Evidence that lactation duration influences incident type 2 diabetes after GDM pregnancy is based on one retrospective study reporting a null association. The Study of Women, Infant Feeding and Type 2 Diabetes after GDM pregnancy (SWIFT) is a prospective cohort study of postpartum women with recent GDM within the Kaiser Permanente Northern California (KPNC) integrated health care system. The primary goal of SWIFT is to assess whether prolonged, intensive lactation as compared to formula feeding reduces the 2-year incidence of type 2 diabetes mellitus among women with GDM. The study also examines whether lactation intensity and duration have persistent favorable effects on blood glucose, insulin resistance, and adiposity during the 2-year postpartum period. This report describes the design and methods implemented for this study to obtain the clinical, biochemical, anthropometric, and behavioral measurements during the recruitment and follow-up phases.

**Methods:**

SWIFT is a prospective, observational cohort study enrolling and following over 1, 000 postpartum women diagnosed with GDM during pregnancy within KPNC. The study enrolled women at 6-9 weeks postpartum (baseline) who had been diagnosed by standard GDM criteria, aged 20-45 years, delivered a singleton, term (greater than or equal to 35 weeks gestation) live birth, were not using medications affecting glucose tolerance, and not planning another pregnancy or moving out of the area within the next 2 years. Participants who are free of type 2 diabetes and other serious medical conditions at baseline are screened for type 2 diabetes annually within the first 2 years after delivery. Recruitment began in September 2008 and ends in December 2011. Data are being collected through pregnancy and early postpartum telephone interviews, self-administered monthly mailed questionnaires (3-11 months postpartum), a telephone interview at 6 months, and annual in-person examinations at which a 75 g 2-hour OGTT is conducted, anthropometric measurements are obtained, and self- and interviewer-administered questionnaires are completed.

**Discussion:**

This is the first, large prospective, community-based study involving a racially and ethnically diverse cohort of women with recent GDM that rigorously assesses lactation intensity and duration and examines their relationship to incident type 2 diabetes while accounting for numerous potential confounders not assessed previously.

## Background

Approximately 7% of all pregnant women are diagnosed with gestational diabetes mellitus (GDM) and comprise a high-risk group for future development of type 2 diabetes mellitus. Women with GDM are 7 times more likely to develop type 2 diabetes after pregnancy [1], although a 4-fold higher incidence of overt diabetes after GDM pregnancy was reported by Gunderson et al. after excluding women with hyperglycemia before pregnancy based on prepregnancy blood glucose measures [2].

About 5-10% of women will be diagnosed with type 2 diabetes within the first 6 months after GDM pregnancy and another 10-15% will develop diabetes within the subsequent 1-2 years postpartum [3-6]. Predictors of diabetes among women with a history of GDM include maternal antepartum and early postpartum glycemia, insulin use during pregnancy, pancreatic β-cell compensation for higher insulin resistance and GDM recurrence [6] and family history of diabetes, especially having a mother with diabetes [7,8]. Prepregnancy obesity, gestational weight gain, postpartum weight gain, and subsequent pregnancies have been associated with higher risk of diabetes years later [6,7,9-15]. In cross-sectional studies, greater central obesity has been reported in women who developed type 2 diabetes after GDM pregnancy [11,12,16].

Lactation intensity and duration have rarely been assessed in relation to type 2 diabetes after GDM pregnancy. Of 28 studies cited in a comprehensive review by Kim et al.[6] and 5 subsequent studies [9,12,13,17,18], only 5 of 33 studies examined lactation status (yes or no) in relation to incident diabetes, and the findings were inconclusive [16,18-21]. Most studies examined "any" lactation versus none, have utilized primarily retrospective designs, involved Latinas, did not conduct standardized postpartum screening for diabetes, and had relatively small sample sizes. Of the only two prospective studies that examined lactation duration in relation to incident diabetes, a previous history of GDM was not ascertained [18,22]. These two studies, including either white or Chinese women, reported that increasing lactation duration was associated with lower incidence of diabetes after pregnancy, which was ascertained via self-report in mid to late life. A retrospective cohort study of White women with a history of GDM found a null association between lactation duration and incident diabetes ascertained by self-report [18]. A major limitation of these three previous studies is that they did not conduct periodic standardized screening of women to ascertain diabetes incidence after pregnancy.

One of the few prospective studies to examine women of reproductive age (50% Black and 50% White) during a 20 year period (1985-2005) is the U.S. multi-center study, the Coronary Artery Risk Development in Young Adults (CARDIA) Study. In CARDIA women, glycemia was measured both before pregnancy and post-weaning to assess the association between lactation duration and incidence of the metabolic syndrome in women with and without previous GDM pregnancies. Longer duration of lactation was associated with a 50-89% reduction in incident metabolic syndrome on average 8 years after pregnancy among women with a history of GDM as well as those with no history of GDM [23]. To our knowledge, lactation intensity has not been evaluated in any previous studies that examined incident diabetes after GDM pregnancy.

We herein critically review the epidemiologic evidence and biological plausibility that lactation may protect women from developing type 2 diabetes in mid to late life. We examine findings from studies of lactation and persistent changes in biochemical risk factors, as well as incident metabolic disease, including type 2 diabetes, after pregnancy. The evidence for short-term changes in metabolic risk profiles and limited evidence from large epidemiologic studies were the impetus for the funding and design of the SWIFT study of postpartum women with recent GDM pregnancy.

The goal of the SWIFT study is to prospectively examine lactation intensity and duration in relation to incident diabetes after GDM pregnancy. The SWIFT study specific aims, design, and methodologies are presented as well as a description of the participant eligibility criteria. We also summarize the recruitment, in-person study assessments and retention protocols for SWIFT, a prospective postpartum cohort of women with recent GDM who delivered a term infant within the Kaiser Permanente Northern California (KPNC) integrated healthcare system.

### Physiological effects of lactation on metabolic status: biological plausibility

Lactation has favorable effects on maternal metabolism including increased glucose-disposal rates, enhanced lipolysis and diverting glucose (> 50 g/d) for utilization in milk production [24-26]. Data are less available regarding whether lactation protects β-cell function [27,28], or has lasting effects on maternal glucose tolerance to ultimately influence the risk of diabetes after GDM pregnancy. Prospective studies are needed that assess lactation more precisely and completely in relation to changes in oral glucose tolerance and body adiposity to determine conclusively whether lactation may delay or prevent future diabetes.

### Lactation and glucose homeostasis

Overall, alterations in the hormonal milieu and responsiveness during lactation are designed to favor lower insulin levels as a result of higher glucose utilization by the mammary gland [25] and increased lipolysis to accommodate the metabolic demands of milk production. Lactation is characterized by increased maternal basal metabolic rates, greater energy needs for milk production, and mobilization of fat stores [25,29,30]. Lactating women generally exhibit lower blood glucose and insulin concentrations and higher glucose production rates due to increased glycogenolysis (not gluconeogenesis or increased use of free fatty acids) [31]. In other cross-sectional studies, lactating women had lower fasting plasma glucose and insulin levels [32], and lower post-absorptive insulin levels than non-lactating women [26].

Frequently sampled oral glucose tolerance (FSOGT) tests showed a higher corrected insulin response at 30 min (*p *< 0.03) in non-lactating (1.24 + 0.26 μU· mg-2 ·102) than lactating women (0.67 + 0.11 μU· mg-2 ·102). Thus, basal and glucose-stimulated β-cell secretory activity for a standardized glucose load may be lower for lactating than non-lactating women [27], indicating that lactation may reduce the load on the β-cells as reported in studies that examined women without glucose tolerance during pregnancy as outlined above.

### Lactation's effects on maternal body weight

About 4-6 kg of body fat is stored during pregnancy partially in preparation for fetal growth during late gestation and lactation [33,34]. In small clinical studies, average weight loss during the first 6 months of lactation in affluent populations is about -0.5 to -0.8 kg/month [35]. Although lactation increases total energy expenditure by 15-25% for milk production [25,36], evidence is inconsistent as to whether lactation promotes greater postpartum weight loss [37].

Prospective studies that measured maternal weights before or during early pregnancy reported lower postpartum weight retention, more rapid return to prepregnancy weight or greater weight losses within 6 months to 1 year postpartum among lactating women [30,38-41]. Greater frequency of lactation and higher breast milk energy output are associated with greater weight loss from 3 to 6 months. Higher intensity of breastfeeding from 2.5 to 6 months postpartum [39,42] and for the first year [43] resulted in 2 kg greater average maternal weight loss. Another study among women (n = 110) classified as fully breastfeeding, partly breastfeeding, or bottle-feeding at six different points in time found that women who lactated more than 1 year lost 2 kg more by 18 months postpartum than women who bottle-fed [43]. Olson et al. in 2003 [38] reported 1.2 kg lower postpartum weight retention in women still breastfeeding at 1 year postpartum, after controlling for first trimester weight and other confounders. More recently, an analysis with a large sample of mothers (n = 32, 920) enrolled in the Special Supplemental Nutrition Program for Women, Infants and Children (WIC) resulted on a modest but significant effect of lactation on weight retention from one pregnancy to the next [44]. Thus, after adjusting for potential confounder effects, lactation for 20 or more weeks resulted on 0.39 kg less weight retention at the beginning of the second pregnancy compared to no lactation. Studies that carefully assessed lactation exposure demonstrate that greater intensity and duration of lactation promote greater postpartum weight loss.

### Overall and regional adiposity during lactation

During lactation, fat stores are mobilized to a greater extent from the trunk and thighs [45-48]. Lactating women show greater declines in suprailiac and subscapular regions but fat increased in the triceps region [35,49]. However, skinfold thickness is a relatively imprecise measure for evaluating total body fat or regional fat, and does not precisely assess changes due to fluid losses [30,41,47].

In studies using dual-energy X-ray absorptiometry (DXA) to measure changes in body composition and regional fat distribution in lactating and non-lactating women, total fat mass showed a greater linear 12-month decline in lactating women versus non-lactating women with the largest decline between 3 and 6 months [35]. No differences in fat mobilization from leg, arm and trunk regions were found, but lactating women had a non-significant 2 kg greater decline in total fat mass [35]. However, this study lacked statistical power to detect this clinically significant difference. Central adiposity is of greater importance metabolically than overall obesity since intra-abdominal (visceral) fat is associated with development of obesity-related insulin resistance and progression to type 2 diabetes [50]. Visceral fat is more metabolically active and is thought to differ from sub-cutaneous fat in the production of adipocytokines that may regulate insulin sensitivity [51].

**Figure 1:**
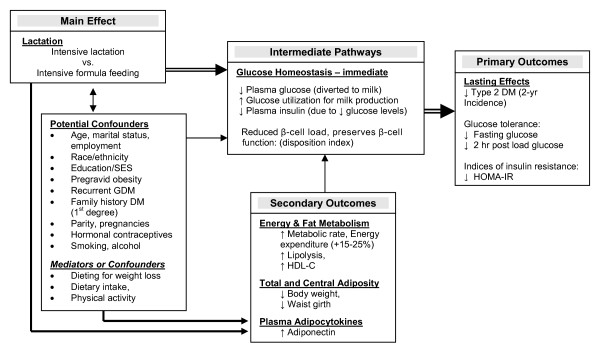
**Theoretical model of effects of lactation on glucose tolerance and Type 2 DM**.

Figure 1 presents our theoretical model and summarizes the hypothesized relationships of lactation to immediate and long-term effects on glucose tolerance as discussed in the preceding sections.

### Lactation and postpartum metabolic status in women with a history of GDM

McManus et al. administered a frequently sampled intravenous glucose tolerance test (FSIGT) to 14 lactating and 12 non-lactating women with previous GDM at 3 months postpartum, who were matched for age, weight, postpartum weight loss and exercise habits. In lactating women, insulin sensitivity, glucose effectiveness and first phase insulin response to glucose (AIRg) assessed by Bergman's Minimal Model were higher, but statistical significance was not reached given the small sample size. However, the disposition index (DI = insulin sensitivity multiplied by AIRg) was 2.5 times higher (129.9 ± 26.0 vs 53.4 ± 18.0 × 10(-4) min(-1); *p *< 0.05) in lactating versus non-lactating women [28]. The higher DI supports the hypothesis that lactation promotes much better β-cell compensation for insulin resistance, which may help maintain β-cell function in the long-term. Because glucose is diverted for milk production, reduced plasma glucose levels "unload" the β-cells such that β-cell function is preserved; insulin response for given levels of resistance is improved. These physiologic changes may sustain glucose tolerance and protect against β-cell exhaustion leading to type 2 diabetes.

Lactation is associated with more favorable glucose metabolism at 2-3 months postpartum in some, but not all small clinical studies. Fasting plasma glucose and insulin levels and incremental responses of plasma glucose (at 30, 60, 90 and 120 min) to a test meal did not differ between lactating (n = 12) and non-lactating (n = 7) groups, but lactating women had lower plasma insulin response and higher rates of glucose utilization [52]. No significant differences in plasma glucose and insulin levels after a 50-gram OGTT were found between lactating and non-lactating groups in two other studies [27,32]. However, the β-cell secretory response assessed by the corrected insulin response at 30 min (CIR 30 min) during the OGTT was reduced by half for lactating versus non-lactating women [0.67 ± 0.11 vs. 1.24 ± 0.26 μU· mg-2 ·102] [27].

A series of cross-sectional and follow-up studies of Latinas with previous GDM examined early postpartum lactation with conflicting findings. Lactation was associated with lower prevalence of diabetes and better glucose tolerance at 4-12 weeks postpartum; a lower total area under the glucose tolerance curve (AUC) (17.0 ± 4.2 vs. 17.9 ± 5.0 g.minute/dL), and lower fasting serum glucose (93 ± 13 vs. 98 ± 17 mg/dL) and 2-hour OGTT glucose levels (124 ± 41 vs. 134 ± 49 mg/dL) after controlling for body mass index (BMI), maternal age and insulin use during pregnancy [19]. Lactation status at 4-16 weeks postpartum was not associated with risk of type 2 diabetes within 5 years [20]. Buchanan et al. examined 122 Latinas with normal fasting glucose and no insulin use during GDM pregnancy and found that those diagnosed with diabetes within 6 months postpartum were less likely to have breastfed (42%) than those with normal glucose tolerance (71%) [16]. Finally, among 91 Latinas receiving OGTT screening at 15-month intervals, lactation status (yes vs. no) at 11-26 months postpartum did not influence onset of type 2 diabetes [21]. Other confounders such as employment, intentional dieting, dietary intake, and physical activity have not been examined in any of the studies. Weight gain and higher BMI during young adulthood are associated with insulin resistance and greater risk of type 2 diabetes [53-55]. No studies have examined changes in waist girth or central adiposity after GDM pregnancy.

### Inconclusive evidence for lasting effects on future disease

Clinical and epidemiologic evidence support the hypothesis that lactation has immediate favorable effects on maternal glucose tolerance, and may reduce the load on the β-cells by lowering plasma glucose levels through diversion of glucose for milk production, thereby lessening insulin demands. The Nurses Health Study (NHS) found a lower incidence of self-reported diabetes by 14-15% for each year of lactation, and a stronger risk reduction for exclusive lactation. This association was independent of current BMI and behavioral risk factors, and there was no interaction by parity. However, the association was null among women with history of GDM, in whom lactation and other lifestyle behaviors (i.e., diet or exercise) were unrelated to diabetes risk [18]. By contrast, a subsequent 20-year longitudinal study based on biochemical measurements before pregnancy and after weaning, found that lactation for 2 or more months versus less than 1 month was associated with 2 to 8-fold lower incidence of the metabolic syndrome after GDM pregnancy controlling for pre-pregnancy risk, socio-demographics, behavioral changes, and race [23].

Evidence is equivocal that lactation, except for exclusive lactation for several months, promotes greater weight loss during the first year postpartum. Few studies have included diverse racial and ethnic groups, assessed postpartum behaviors, weight loss, regional adiposity, or other factors in relation to long-term maternal glucose tolerance after GDM pregnancy.

Lactation has immediate favorable effects on glucose tolerance, but only limited evidence for any long-term effects. Studies have never prospectively assessed both intensity and duration of lactation as well as other major confounders in women with recent GDM. Well-controlled, prospective studies with more precise and complete measures of lactation are required to assess the impact on development of type 2 diabetes. Data are currently unavailable to conclude that lactation reduces the risk of type 2 diabetes after GDM pregnancy.

The Diabetes Prevention Program (DPP) showed a greater than 50% reduction in the incidence of diabetes with a weight management/exercise program for high risk adults, demonstrating that a relatively modest weight loss of 3-4 kg (equivalent to 5% of initial body weight) could prevent diabetes [56]. A relatively modest 2 kg higher weight loss within 1 year has been attributed to intensive lactation in the studies that prospectively assessed lactation intensity and measured body weights. Based on the DPP findings, lactation may lower the incidence of diabetes by 30% after GDM pregnancy based on loss of fat mass. If lactation exerts other effects (e.g., preservation of β-cell function) that are independent of adiposity, then the reduction in type 2 diabetes may be even greater.

The American Academy of Pediatrics recommends breastfeeding as the preferred method of infant feeding for at least 1 year of age [57]. Although 80% of U.S. women initiate lactation, only 45% report "any" level of breastfeeding at 6 months [57]. Lactation is a modifiable behavior that may be translated into a practical, low-cost intervention, and has the potential to enhance postpartum interventions that have primarily relied on strategies to promote healthy diet and increase physical activity levels. Breastfeeding may prevent recurrence of GDM in a future pregnancy, and thereby also influence the risk of type 2 diabetes in the offspring [58-61].

### The SWIFT study design and aims: prospective GDM cohort

The overall objective of this study is to assess whether lactation prevents the onset of type 2 diabetes during the first 2 years postpartum among women with recent GDM, after taking into account their age, race/ethnicity, parity, weight status, education, severity of gestational glucose intolerance, GDM recurrence history, family history of diabetes, clinical or medical risk factors, and other postpartum behaviors.

Specifically, we aim to determine whether intensive lactation compared with intensive formula feeding is associated with:

Lower 2-year incidence of type 2 DM (Aim 1);

Lower fasting and 2-hour post-load plasma glucose levels, and lower insulin resistance (Aim 2); and

Lower total and central adiposity, and higher plasma adiponectin levels (Aim 3).

Another objective of the study is to determine whether lower adiposity is associated with lower incidence of type 2 diabetes in this population (Aim 4).

## Materials and methods

### Study design and setting

The study population is recruited from members of the KPNC integrated healthcare system who received prenatal care and delivered at a KPNC hospital. The demographic profile of KPNC membership is representative of the diverse racial and ethnic groups in the same geographical area [62].

The study design is a prospective cohort of women who were diagnosed with GDM before 34 weeks gestation. During the study period, GDM diagnosis is based on standardized 3-hour 100 g oral glucose tolerance tests during pregnancy using Carpenter and Coustan's criteria [63] as recommended by the American Diabetes Association (ADA) [64]. Two or more of the four plasma glucose values have to meet or exceed the plasma glucose thresholds recommended by the ADA [64] and the American College of Obstetricians and Gynecologists (ACOG) [65], and received standard treatment for GDM within by KPNC prenatal care providers.

Standard obstetrical practice at KPNC involves postpartum follow-up screening for diabetes in women with GDM via the 2-hr 75-gram OGTT at 6-9 weeks postpartum as recommended by the ADA [66]. As part of this SWIFT study, we conduct the follow-up OGTT during the baseline study visit, perform in-person interviews, and obtain anthropometric measurements during the 2 h period. Women who are free of diabetes at the baseline visit are screened for incident diabetes annually for 2 years, and undergo assessments of other parameters.

The National Institutes of Health provided funding to conduct the study (5-years from the National Institute of Child Health and Human Development, R01 HD050625). The study protocol was approved by the Institutional Review Board at Kaiser Permanente Northern California.

### Study sample

#### Study cohort

The cohort is being recruited from 13 KPNC medical centers and medical office facilities through the 5, 000 square mile KPNC region. Participating sites within the three areas include: North area: Sacramento, South Sacramento, and Roseville Medical Centers, and Rancho Cordova, Elk Grove, Point West, and Folsom Medical Offices; East area: Division of Research (DOR) Research Clinic (Oakland), Hayward and Richmond Medical Center; South area: Fremont, Santa Clara, and San Jose Medical Centers. The cohort includes women who received prenatal care and delivered a singleton, live born infant at a KPNC hospital between July 2008 and October 2011.

#### Eligibility criteria

Age 20-45 years at delivery,

Availability of clinical medical record and delivery record from the KPNC Health Connect electronic medical record,

GDM pregnancy diagnosed by Carpenter and Coustan's criteria,

Delivered a singleton, live birth ≥ 35 weeks gestation,

No pre-existing diabetes or other serious medical conditions prior to index GDM pregnancy,

No diabetes diagnosis at 6-9 weeks postpartum for the index GDM pregnancy,

No use of steroids, or other medications significantly affecting glucose tolerance,

Not planning to move from the northern California area within the subsequent 2 years,

Not planning another pregnancy within the next 2 years, and

English or Spanish speaking.

Eligibility is also based on infant feeding practices, infant feeding intentions and status at 6-9 weeks postpartum:

*Intensive formula feeding*: did not breastfed or provided at least 14 oz of formula per day during the first 4 months postpartum.

*Intensive lactation*: only breast milk or no more than 6 oz/day of formula supplementation within 6-9 weeks postpartum, and intention to continue breastfeeding intensively for at least 4 months postpartum.

#### Sample size and power calculation

The target recruitment sample is 1, 098 women with recent GDM who are free of type 2 diabetes at 6-9 weeks postpartum, with the expectation of having them equally distributed into the two infant feeding groups (i.e. intensive formula feeding, and intensive lactation groups).

Based on a previous study of pregnant women at KPNC [67] and on KPNC clinical data, we expect 89.8% of the sample to remain in analyses of the 1-year exam (N = 986), and 78.5% to remain in analyses of the 2-year exam (N = 862).

For assessing Aim 1, assuming a two-year postpartum cumulative incidence of type 2 diabetes of approximately 20% as described in our KPNC GDM population [68], and given the expected censoring rates above, we expect to observe approximately 172 incident cases of type 2 diabetes. We will have sufficient power (0.80) to detect a relative hazard of type 2 diabetes associated with intensive lactation of 0.65 (Table 1).

**Table 1:** Minimum detectable relative hazard of incident Type 2 diabetes mellitus and minimum detectable differences in mean change in biochemical measures^a^

Outcomes	Relative Hazard; 2-year Cumulative probability of Incident Type 2 DM = .20	Detectable Difference (SD units) in Mean change 1-year, N_1 _= N_2 _= 493	Detectable Difference (SD Units) in Mean change 2-year, N_1 _= N_2 _= 431
**Infant Feeding Categories**			
Intensive formula feeding	(referent)	(referent)	(referent)
Intensive lactation (4 or more months)	0.65	0.179	0.191

^a ^Standard deviation units, two-sided test, significance level = 0.05, power = 0.80.

We also estimated minimum detectable absolute differences in mean change from baseline in a continuous variable, expressed in standard deviation units, at the year 1 and year 2 exams (relevant to Aim 2 and 3). Based on published estimates [39,56] of standard deviations of 1 year change, we will have sufficient power to detect the following mean changes in the outcomes across infant feeding categories at year 1: plasma glucose and insulin, respectively, 0.45 mg/dl and 0.27 μU/ml at fasting, and 1.69 mg/dl and 3.38 μU/ml at 2-hr post load, and postpartum weight 0.25 kg and waist girth 0.28 cm.

To test Aim 4, we assumed a graded linear trend in relative hazards across quartiles of adiposity and plasma adiponectin measures. Power calculations are conservatively based on a global test for association by treating the risk factor as a categorical variable in the Cox model (i.e., testing a set of indicator variables rather than a test for linear trend) (Table 2).

**Table 2:** Minimum detectable relative hazards of Incident Type 2 Diabetes Mellitus associated with quartiles of adiposity and adiponectin^a^

Quartiles	Relative Hazard (> 1; adiposity)	Relative Hazard (< 1; adiponectin)
**Q1**	(referent)	(referent)

**Q2**	1.26	0.79

**Q3**	1.58	0.63

**Q4**	1.98	0.50

^a^Two-sided test, significance level = 0.05, power = 0.80

Since Aim 2 and 3 will be assessed via linear models with adjustment for multiple confounding, we calculated minimum detectable additional (or incremental) proportions of variance in the dependent variable explained by infant feeding practices (denoted ΔR^2^) over and above that already explained by a given set of confounders (denoted R^2^). Assuming a range in R^2^ of 0.05 -0.30, a power of 0.80 and a 10-parameter model, minimum detectable incremental variance in outcome explained by infant feeding practices (intensive lactation vs. intensive formula feeding) ranges from 0.55% to 0.75%, depending on the explanatory power of confounding variables included in the model (Table 3; calculations presented are for the one-year visit).

**Table 3:** Minimum detectable increases in the proportion of variance in continuous variables explained by infant feeding^a, b, c^

Variable tested	Degrees of Freedom for Test Variable	R^2 ^for confounders			
		
		0.05	0.10	0.20	0.30
Infant feeding (intensive lactation vs. intensive formula feeding)	1	0.0075	0.0071	0.0063	0.0055

^a^Continuous variables: plasma glucose and insulin levels, indices of insulin secretion, body weight or waist girth, and adiponectin

^b^Adjusting for confounders that account for various proportions of explained variance (R^2^)

^c^N = 986 (one-year exam), significance level = 0.05, power = 0.80

### Study procedures

**Figure 2:**
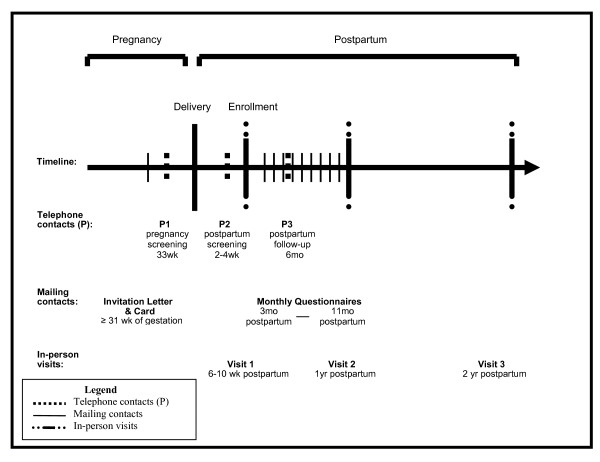
**Schedule of study contacts and assessments**.

Eligibility screening and data collection activities for this longitudinal cohort study include three telephone contacts, three in-person study visits and 10 mailing contacts from late pregnancy through 2 years postpartum (Figure 2). Recruitment is expected to be completed in December 2011.

#### Eligibility pre-screening activities

Once a week a list of women recently diagnosed with GDM in one of the 13 study sites is populated into a Tracking System (TS) specially created for the study. Authorized study staff reviews the electronic medical records of the women listed to verify language preference, eligibility information, if available (i.e. singleton vs. multiple pregnancy and estimated delivery date), and primary KPNC clinical care provider's name.

KP providers are contacted via email to request authorization to contact their patients. Passive approval is assumed after 2 weeks as explained in the email letter. An invitation letter describing the study is sent to potential participants along with a pre-paid postage, addressed refusal return card to indicate whether they want or not to be contacted by the study staff and the best way to be reached.

Around 33 weeks of gestation, study staff call potential participants to describe the study and administer a pre-screening questionnaire asking about moving, pregnancy and infant feeding plans using a standardized scale [69,70], as well as other eligibility criteria (e.g. pre-existing diabetes, singleton vs. multiple gestation).

#### Screening phone call

Once a week the TS is populated with delivery information (i.e. singleton, live birth > = 35 weeks of gestation, birth weight and length and Apgar score) from KPNC electronic medical databases for potential participants who gave birth, which allows to confirm eligibility for further contacts.

Around 2-4 weeks postpartum, potential participants are contacted by phone to determine their interest and eligibility to participate in the study. At the telephone screening contact, a screening questionnaire similar to the one administered during late pregnancy is completed; additionally, infant feeding practices are assessed to determine potential eligibility as described below. Eligible women are invited to participate in the study and scheduled for their baseline study visit at 6-9 weeks postpartum.

#### Study visits

Study data collection occurring at in-person visits to KPNC clinics is scheduled at the following postpartum time points: Visit 1 at 6-9 weeks (baseline), Visit 2 at 12 months and Visit 3 at 24 months postpartum. The study visits take place at the 13 KPNC clinical facilities where we are approved to implement the study protocol and conduct visits.

Signed informed consent for participating in the study is obtained at the baseline in-person visit which includes permission to obtain information on perinatal outcomes from electronic databases.

Study procedures at each visit include anthropometric measurements, self- and interviewer-administered questionnaires to collect data on socio-demographics, clinical (reproductive and medical history, depression), and early postpartum behavioral characteristics (infant feeding practices, smoking and alcohol consumption, dietary and caffeine intake, physical activity and sleeping patterns), and administration of the 2-hour, 75-gram OGTT and collection of blood specimens (fasting and post load). Diagnosis of diabetes requires confirmation by a second laboratory test on another day. Women who are free of type 2 diabetes at baseline are followed prospectively, while women who are confirmed with diabetes are informed via a study letter and recommended to contact their health care provider for treatment and education.

Spanish versions of the consent form and each questionnaire are available for participants whose preferred language is Spanish.

#### Mailing questionnaires

After the baseline study visit, participants begin receiving a monthly mailing questionnaire with a pre-paid postage, addressed return envelope (non-respondents are contacted by phone) from 3 through 11 months postpartum which assesses lactation status and use of milk supplements and other infant foods during the previous week. Additional questions about contraceptive use, pregnancy status and recent diabetes diagnosis are also included in the questionnaire.

#### Phone follow-up interview

At 6 months postpartum, a 20-minute follow-up phone interview is conducted to collect data on maternal and child health (e.g. health problems, medications, recent diabetes diagnostic), pregnancy status and contraceptive use, socio-demographic information (school attendance and maternal employment status) and infant feeding history.

A subset of women and their children are enrolled in an ancillary study (SWIFT Offspring Study) which involves attending a study visit at 6 months postpartum; for those women the option to complete the six-month questionnaire at that visit (instead of doing it over the phone) is offered.

### Assessment of the main exposure of the study

#### Lactation intention assessed in late pregnancy

We assess lactation intentions using the Infant Feeding Intentions (IFI) scale developed by Nommsen-Rivers and Dewey [69] to quantitatively measure maternal breastfeeding intentions. The first 2 items of the scale measure strength of intentions to initiate breastfeeding and subsequent items assess strength of intentions to be breastfeeding exclusively at 1, 3 or 6 months. The IFI scale provides total scores ranging from 0 ot 16; it has demonstrated strong internal consistency, content and construct validity [69], and it has been used successfully with diverse populations [70].

#### Formula feeding log mailed at 2 weeks postpartum

A formula feeding log is mailed to mothers 2 weeks after delivery on which they are asked to record the amount of infant formula given per bottle and the average number of bottles given at each week postpartum, since birth through week 5. This log is an aid for answering the questions on infant feeding asked by phone at 4 weeks postpartum.

#### Lactation status and intention assessed at the 4-week postpartum telephone call

Potential participants are asked about their infant feeding practices to determine their eligibility. Data collected include information on breastfeeding (ever and current), breast milk expression and weaning. In addition, they are asked about infant formula use, including starting date, number of days that formula was given to the baby and the average amount given per day, both since birth and during the past week. Data on introduction of any other liquids and the amount given are also collected. Intentions to continue breastfeeding until at least 3 or 6 months are explored using two items from the IFI among those who are either giving breast milk only or no more than 6 oz of formula per day. Based on their responses, mixed feeding mothers (50% breast milk and 50% formula) are identified, considered ineligible for the study and therefore not invited to enroll.

#### Lactation status at enrollment (baseline) and follow up visits

At the baseline visit, participants complete an interviewer-administered questionnaire on breastfeeding. Questions include time of milk arrival (lactogenesis stage II), usual breastfeeding frequency during the day and night, typical frequency of breast milk expression and of expressed-breast-milk feeding, weaning date and reasons for not initiating breastfeeding or weaning are also asked.

In addition, mothers are asked about introduction of infant formula or other milk, date it started, kind of formula/milk, usual number of bottles given per day and typical number of ounces the baby drank from the bottle. Similarly, introduction of sweetened water, fruit juice and other liquids is also asked, as well as use of Pedialyte, a common treatment for diarrhea. We also ask them about introduction of any solid foods, starting date, type and average given per day during the past week. Finally, mothers are asked about their reasons for introducing formula.

Similar information about lactation is collected at the follow up visits when the child is about 1 and 2 years of age. Additionally, information on lactation history (i.e. breastfeeding duration per each older child and maternal age at first and last lactation) is collected at the last in-person visit (visit 3).

#### Lactation assessment from 3 through 11 months postpartum

A monthly assessment of lactation status, use of milk supplements and other infant foods during the previous week is done using mailing questionnaires. We ask about baby's age at weaning, breastfeeding frequency (during the day and night), and breast milk expression frequency, age at first formula feeding, formula feeding frequency and ounces per bottle, introduction of any other liquids or solid foods, and age when first given.

At 6 months postpartum, participants are interviewed over the phone to collect similar information on infant feeding practices.

### Outcomes of the study

#### Diagnosis of type 2 diabetes

Diagnosis of incident type 2 diabetes is the primary outcome of the SWIFT study and is defined according to the 1997 ADA criteria [71]: fasting plasma glucose (FPG) > = 126 mg/dL or 2-hour plasma glucose (2-h PG) after a 75-gram oral glucose load > = 200 mg/dL. The diagnosis will require confirmation by a second test according to the same criteria [71]. We determine diabetes status using data obtained from OGTT at 6-9 weeks, 12 months, and 24 months postpartum in all participants.

For women who will have diabetes diagnosed by their physician at follow-up visits, their medical record will be reviewed in order to assess diagnostic plasma glucose values and they will have fasting plasma glucose tested in order to confirm diabetes status according to the study criteria [71].

#### Insulin resistance

Indices of insulin resistance will include the insulin sensitivity index (ISI), quantitative insulin sensitivity check index (QUICKI) and homeostatic method (HOMA-IR) based on glucose and insulin measures. HOMA-IR and QUICKI are strongly correlated (0.6-0.8) with euglycemic-hyperinsulinemic clamp and FSIGT techniques which are the most accurate, direct measures of insulin resistance. We will assess insulin resistance and sensitivity using data obtained from 0 to 120 min during OGTT at 6-9 weeks, 12 months, and 24 months postpartum in the cohort.

#### Body size and central adiposity measurements

Weight and waist circumference will be measured at baseline and each follow-up visit, and height will be measured at baseline. BMI will be calculated as weight (kg) divided by height (m) squared and used to evaluate overweight/obesity or total adiposity. Central adiposity will be assessed by waist girth.

### Other study measurements

#### Anthropometry

Weight and waist circumference are measured at baseline and each follow-up visit. Women are weighed at 12 sites using a portable Tanita WB 100A digital scale, which measures up to 440 lb (or 200 kg). At the 13th site (DOR Research Clinic) we obtain weight using a Tanita Model 3101 Portable Medical Stand-On scale which measures up to 900 lb. Women are measured in light clothing (e.g. no jacket or sweater) and without shoes, after emptying their pockets and removing any accessories that could impact their weight measurement (e.g. cell phones, jewelry, watches or belts). Weight measurements are recorded in pounds with 0.2 lb graduation.

Waist circumference is measured in centimeters to the nearest millimeter using the Gulick II Plus anthropometric tape (Model 67019). This no-stretch, retractable tape with both centimeter and inch gradations, has a tensioning device attached to provide a known amount of tension while a measurement is being taken. Unless the participant refuses, the tape is applied directly on the skin, horizontally at a level laterally that is midway between the iliac crest and the lowest lateral portion of the rib cage and anteriorly midway between the xiphoid process of the sternum and the umbilicus. Two consecutive measurements are taken and recorded, and a third measurement is taken and recorded if the first and second measurements differ more than 1 cm.

Height is measured only at baseline, using a Seca Portable Stadiometer (Model 67029) with a measurement range between 8" and 82" and gradations in inches and centimeters. Height measurements are recorded in centimeters to the nearest millimeter. Participants are asked to remove their shoes and any hair ornaments to get an accurate measurement.

#### Dietary intake

The PrimeScreen [72], a brief self-administered food frequency questionnaire, is being used to assess quality of participants' diet. This dietary tool has demonstrated adequate reproducibility when re-administered after 2 weeks, and its results compared well with two reference standards: the self-administered brief semi-quantitative food frequency questionnaire (SSFQ) by Willet and colleagues [73] and plasma levels of selected nutrients [72].

It consists of 18 questions about average frequency of consumption of specified foods and food groups, and another 7 items about vitamin and supplement intake. The original recall timeframe was the past year; however, for the purpose of this study we used a past week recall timeframe. The five frequency of consumption categories used are: none (we changed the original 'less than once per week' category to make it consistent with the modified recall timeframe), once (originally 'once per week'), 2-4 times (originally '2-4 times per week'), nearly daily or daily, or twice or more per day.

In this study we use the PrimeScreen as an interviewer-administered questionnaire. Cue cards displaying the answer options or pictures of several food items were prepared as supplemental materials. These aids are particularly helpful for interviews in Spanish, given that some foods have different names in different dialects.

#### Caffeine intake

The Supplemental Beverage Questions (^© ^2004 Fred Hutchinson Cancer Research Center) was selected to assess caffeine intake. The questionnaire, which is available to the public at the center's web page, ask about the frequency of consumption of 13 groups of caffeinated and non-caffeinated beverages, as well as the serving size in relation to what is considered a medium serving size (i.e. an 8-ounce cup for coffee or tea, a shot for espresso drinks, and a 12-once can for sodas). The original questionnaire, available on-line, does not indicate a specific recall timeframe; for the purpose of this study, we asked about caffeine intake during the past month.

#### Physical activity

Physical activity is assessed at each study visit using an adapted version of the Pregnancy Physical Activity Questionnaire (PPAQ) developed by Chasan-Taber and colleagues [74]. The PPAQ is a self-administered semi-quantitative questionnaire that asks respondents to report the time spent participating in 32 activities including household/caregiving (13 activities), occupational (5 activities), sports/exercise (8 activities), transportation (3 activities), and inactivity (3 activities). For each activity, respondents are asked to select the duration category that best approximates the amount of time spent in that activity per day or week during the current trimester of pregnancy. Duration categories range from 0 to 3 or more hours per day or week, except for occupational activities with a range from 0 to 6 or more hours per day. The PPAQ has been validated with pregnant women with reproducibility measures from 0.78 to 0.93 depending on the type of activity [74].

The PPAQ was selected under the assumption that postpartum women have similar physical activity patters as pregnant women, but we slightly adapted it for use with non-pregnant women. The recall timeframe was changed to the past week, an item on prenatal exercise class was modified to 'postnatal' exercise class (for the baseline visit) and to 'exercise class' (including space to specify which kind, for the follow-up visits), and an activity expected among women with young children was added as an item (i.e. 'walking, pushing a stroller'). In addition, two other items were modified: a) we added 'walking a dog' to the 'playing with pets' item, and b) we deleted the word 'quickly' from the 'walking quickly up hills for fun or exercise' item. For this study, the questionnaire was interviewer-administered.

#### Sleep questionnaire

The Sleep Questionnaire has two sections: a) general sleep habits and b) the general sleep disturbance scale (GSDS) adapted from a sleep questionnaire for the postpartum period [75]. The General Sleep Habits section was developed for the study and consisted of 6 questions regarding sleep habits during the past week. Information asked includes: get-up and go-to-bed time, time to fall sleep, frequency of waking up in the night, hours of actual sleep at night, minutes of actual sleep from naps. Questions about get up/go to bed time and minutes/hours of actual sleep are asked both, for week days (or work days) and for the weekend (or days off).

The GSDS is used to assess subjective sleep characteristics during the past 7 days. The GSDS is a 21-item, 0-7 rating scale, which includes seven subscales: sleep onset, waking up during sleep, waking up too early from sleep, quality of sleep, quantity of sleep, daytime functioning, and use of substances to help induce sleep [75]. Higher scores indicate more disturbed sleep, and a mean score of 3 on the total GSDS or any of the subscales represents the '3 days per week' DSM-IV criterion for insomnia, which is considered a clinically significant sleep disturbance [75]. The GSDS has been successfully used with postpartum mothers [76,77].

#### Depression

The 20-item Center for Epidemiologic Studies-Depression (CES-D) [78] is used to assess depression symptoms over the past 7 days. The CES-D has been broadly used in epidemiologic research with different racial and ethnic populations in the United States, showing similar reliability and general structure of responses among these groups [79]. This scale is widely used with pregnant and postpartum women populations and provides a consistent assessment at the longitudinal visits [80-82]. A score of 16 indicates high risk for clinical depression [78].

#### Clinical risk factors

Using self- and interviewer-administered questionnaires, we collect information on clinical risk factors at the in-person visits. We ask about family history of diabetes, previous pregnancy GDM diagnosis and other perinatal complications, pre-pregnancy weight, current medical conditions, medication use, pregnancy status after enrollment (intercurrent pregnancies), and hormonal contraceptive use, including type and duration.

Women who develop medical conditions during the study period may continue their participation, except for rare diagnoses of cancer (except for skin cancer), major organ failure (kidney, heart, or liver) or other life-threatening conditions. Women who become pregnant during follow up continue in the study after the delivery of the additional pregnancy. Electronic databases will be used to confirm some of this information.

### Biospecimen procedures and laboratory assays

Venous blood is drawn from participants in a non-pregnant state by a trained phlebotomist at each study visit. A fasting blood sample is drawn after a minimum of 8 h of overnight fasting, followed by consumption of the 75-gram oral glucose solution (within a period of 5 min), and followed by a second blood sample 2 h after the intake of the glucola beverage. Participants are instructed not to smoke, walk or engage in any physical activity, not to chew gum, eat mints or candy, and not to consume any food or beverages during the test, except small sips (3 oz) of plain water. Normally, the participants complete all the other study assessments (i.e. anthropometric measurements and questionnaires) during the 2-hour waiting time. Breastfeeding mothers are instructed to breastfeed their babies and/or express breast milk before the fasting blood sample is drawn, and the frequency and duration of any breastfeeding during the test is recorded. At every visit, 30 mL of blood are drawn into ethylene diamine tetraacetic acid (EDTA) tubes for the fasting blood draw, and 15 mL of blood are drawn for the 2-hour post glucola blood draw for measurement of glucose and insulin.

Blood samples are processed, aliquoted and placed in the freezer within 90 min of collection. Using the internal KP courier system for biospecimens, aliquoted plasma is then transported from the study sites to the KPNC Regional Laboratory and from there to the DOR research clinic for storage at -70°C, in a low temperature freezer maintained and monitored at constant temperature. Upon arrival at the DOR research clinic, cryogenic vials are scanned into the SWIFT biospecimens database. Stored samples for analyses are shipped monthly to the University of Washington, Northwest Lipid Research Laboratories, Immunoassay Core of the Diabetes Endocrinology Research Center (DERC) at the University of Washington, Seattle, Washington (Dr. Santica Marcovina, Director) for analysis of glucose, and insulin.

Analyses of glucose are performed enzymatically on Hitachi 917 Autoanalyzer using the combined catalytic activities of hexokinase and glucose-6-phosphate-dehydrogenase. The assay of total immunoreactive insulin, or total insulin, is performed by a double-antibody radioimmunoassay developed in the Diabetes Endocrinology Research Center Immunoassay Core Laboratory.

### Retrieval of data from KP electronic databases and medical records

Some data will be collected using electronic databases to obtain accurate information and decrease the study time burden on subjects. Pregnancy outcomes data are available in KPNC in-patient and outpatient databases including labor and delivery history, infant birth weight and length, Apgar scores, neonatal intensive care unit admissions, formula intake, medical history, gestational age at diagnosis of GDM, 100-g, 3-hour OGTT results, insulin use during pregnancy, mode of delivery, pregnancy admissions, treatments and complications. Measured gestational weights, information on subsequent (inter-current) pregnancies and other pregnancy complications can also be obtained or confirmed from those electronic databases. Data on hormonal contraceptives during the postpartum period are also available from the pharmacy electronic databases.

### Quality control procedures

Detailed manuals were developed describing data collection procedures. Research staff completes training led by the Project Manager which includes a series of shadowed study activities with each data collector before this person can start performing any study activity and observation when they conduct their first few measurements with actual participants. Initial training for anthropometric measurements involved measuring 5 female adult volunteers following a standard protocol and comparing measurements by the technicians with those of the Project Manager, who had training in nutrition and was considered the gold standard. Throughout the study, refresher trainings involving all data collectors are being conducted twice per year.

A relational database architecture (TS) has been developed in "Access" specifically for this study. The TS was designed to be populated weekly with potential participants (women recently diagnosed with GDM), to evaluate study eligibility, to track participants and to enter study data. It includes all data from prenatal and delivery record abstraction, blood analyses, participant appointments, contact and provider information, screening interviews, study questionnaires and results from any contact with participants or potential participants. Data from the questionnaires are entered by study staff into the TS and transferred to SAS data sets for data cleaning, merging, analysis and reporting. All data are secured from external access through password protection and tracking of access to accounts on the central mainframe, and PC workstations, and from computer equipment failure by daily incremental back-up and off-site archival.

Different strategies are in place to increase participation and retention. The annual OGTT testing for type 2 diabetes is not routinely done at KPNC and is presented as a good reason to participate. The study provides participants with a copy of their OGTT results to give to their primary care provider. Women are allowed to bring their infants to the study visits, and are given a light breakfast after the 2-hour blood draw. Incentives for participating include: 1) a $60 gift card for attending and completing a study visit, 2) a waiver of any co-pay for the OGTT performed at each study visit, 3) parking validation for attending a study visit, if applicable, 4) additional small gifts for attendance to each visit, such as bibs, 5) a $15 gift card for completing and returning 6 (out of 9) monthly mailed questionnaires during the first year of participation.

Retention is maximized by mailings of reminder letters to all participants scheduled for a study visit; text messages and/or emails are sent before each study visit. In addition, contact information is confirmed at any in-person, phone or mail contact.

## Discussion

SWIFT is the first study to prospectively examine whether lactation intensity and duration are associated with a lower the 2-year incidence of type 2 diabetes after GDM pregnancy, controlling for multiple potential confounders. However, as an observational study, it has some specific limitations. Because women are not randomly assigned to infant feeding groups there is a chance for unequal distribution of confounders among the exposure groups and inherent differences due to their selection of the specific behavior. To address this potential for bias, we measure as many as possible known potential confounders (i.e., lifestyle behaviors), and adjust for pertinent ones in the analysis. We plan to analyze our results controlling for potential confounding from clinical and postpartum behavioral and clinical risk factors that have not been assessed in previous studies.

Another limitation relates to the indices of insulin resistance, HOMA-IR and QUICKI, which are not as accurate as the euglycemic-hyperinsulinemic clamp or FSIGT to measure insulin sensitivity/resistance. The indices of insulin resistance have been widely used in population-based studies and are good predictors of future diabetes. The clamp and FSIGT methods are not feasible for large epidemiologic studies, especially with repeated measurements because the technique is time consuming, invasive and expensive. Postpartum women without any overt disease are unlikely to participate in such invasive procedures.

A particular advantage of SWIFT relates to its population within the KPNC integrated healthcare system setting. The racial/ethnic diversity of the KPNC population increases our chances to obtain a representative sample and permit generalizability of the findings; also the high retention observed (> 80%) in previous KPNC studies is an important goal in cohort studies. Furthermore, the accessibility of patient's data from the KPNC electronic medical records will lessen subject burden and allow for confirmation of self-reported data.

Moreover, the SWIFT study will be able to evaluate short-term effects of lactation on maternal glucose homeostasis and adiposity which will provide data on possible mechanism to explain any association between lactation and incidence of type 2 diabetes during the follow-up period.

If lactation is found to have persistent effects on maternal glucose homeostasis that prevent type 2 diabetes in women, then translation of the findings from the SWIFT may have a significant public health impact. Lactation promotion would be a low-cost, feasible strategy that may enhance postpartum behavioral interventions for the prevention of diabetes in women.

## Competing interests

The authors declare that they have no competing interests.

## Authors' contributions

All authors contributed to the overall study design and specific methodologies. KGD participated in the design of lactation assessment. JVS participated in the diabetes diagnosis protocol. AF participated in gestational diabetes ascertainment. BS participated in the physical activity assessment. JCL participated in the development of the biospecimens protocol. CPQ performed sample size calculations and developed the data analysis plan. SRH participated in the study coordination. SLM participated in the design of lactation assessment and drafted sections of the manuscript. EPG conceived the study design, oversaw the implementation, and drafted sections of the manuscript. All authors read and approved the final manuscript.

## Pre-publication history

The pre-publication history for this paper can be accessed here:

http://www.biomedcentral.com/1471-2458/11/952/prepub
